# A Novel MicroRNA From the Translated Region of the *Giardiavirus rdrp* Gene Governs Virus Copy Number in *Giardia duodenalis*

**DOI:** 10.3389/fmicb.2020.569412

**Published:** 2020-11-23

**Authors:** Pengtao Gong, Xianhe Li, Wei Wu, Lili Cao, Panpan Zhao, Xin Li, Baoyan Ren, Jianhua Li, Xichen Zhang

**Affiliations:** ^1^Key Laboratory of Zoonosis Research, Ministry of Education, College of Veterinary Medicine, Jilin University, Changchun, China; ^2^Jilin Academy of Animal Husbandry and Veterinary Medicine, Changchun, China

**Keywords:** *Giardia duodenalis*, *Giardiavirus*, microRNA, coding region, rdrp gene

## Abstract

*Giardia duodenalis* is an important zoonotic parasite that can cause human and animal diarrhea. *Giardiavirus* (GLV) is a double-stranded RNA virus in Totiviridae family, which specifically infects trophozoites of the primitive protozoan parasite *G. duodenalis.* However, the GLV infectious and the pathogenicity of the *G. duodenalis* still remain to be confirmed. The GLV genome is 6,277 bp, which encodes two proteins (Gag and Gag-Pol). The expression of Gag-Pol protein is regulated by a-1 ribosomal frameshift. In this report, we identified a novel microRNA (GLV miRNA1) from the GLV. Split ligation northern results showed that GLV miRNA1 is a special expression product of GLV, and the precursor was also identified by primer extension. Antisense sequence of the GLV miRNA1 could increase the copy number of virus in *G. duodenalis*. It suggests that GLV miRNA1 governs the copy number of *Giardiavirus* in *G. duodenalis*. Most importantly, the GLV miRNA1 lies at the translated region of the *rdrp* gene, which is the first case that microRNA locates in the translated region of a known protein. It may be implying a novel phenomenon for miRNA biogenesis.

## Introduction

The discovery of regulatory small RNAs has reshaped the paradigms in both molecular biology and virology (Perez et al., 2010). MicroRNAs (miRNAs) are a class of endogenous small non-coding RNA molecules, which regulate numerous and diverse eukaryotic cellular processes (Reinhart et al., 2000; Aguilar et al., 2019), including virus life cycles (Sullivan et al., 2005; Verna and Karyn, 2020). The canonical miRNA biogenesis pathway is that miRNA genes are first transcribed into primary miRNAs (pri-miRNAs) using RNA polymerase II. Next, pro-miRNAs with a stem–loop containing structures are processed via RNase III enzyme of double-stranded RNA-specific endoribonuclease nuclear type III (*Drosha*) and microprocessor complex subunit DiGeorge syndrome critical region 8 (DGCR8) within nucleus and then cleaved into smaller double-stranded RNA of pre-miRNAs. Following, pre-miRNAs are exported to the cytoplasm with the aid of exportin 5, cleaved by the RNase III enzyme cytoplasmic endoribonuclease with dsRNA “dicing” activity (DICER). Mature miRNAs are generated by unwinding the double strands via an RNA-induced silencing complex (RISC). Non-canonical miRNA biogenesis pathway is Drosha or DGCR8 independent, and miRNA genes are processed to pre-miRNAs by the spliceosome machinery (Kincaid and Sullivan, 2012; Jesús et al., 2013). Interestingly, some viruses also encode their miRNAs, which are termed as viral miRNAs. The first viral miRNA was identified in Epstein-Barr virus (EBV) (Pfeffer et al., 2004), and all the EBV-encoding miRNAs locate in untranslated region (UTR) or intronic region of open reading frame. To date, more than 500 viral miRNAs have been reported (Kozomara and Griffiths-Jones, 2011). Most of them could target to the host immune system genes (Stern-Ginossar et al., 2007; Naqvi et al., 2018; Murer et al., 2019). Plenty of viral miRNAs were found in the herpesvirus family, which has long DNA genomes (Sorel and Dewals, 2016). Thus, it gave us a chance to explore the cross-talk between virus and host in viral infections and pathogenesis. MiRNAs encoded by Simian virus 40 (SV40) could be secreted by exosomes to target the immune-regulatory gene of host immune cell (Pegtel et al., 2010) to enhance the probability of successful infections (Sullivan et al., 2005). Initially, naturally occurring RNA viruses that express miRNAs are not widely accepted. It had been speculated that viruses with RNA genomes would not encode miRNAs due to negative effects on fitness that would be incurred with *cis* cleavage of the genome, antigenome, or mRNAs mediated by the miRNA processing machinery (Kincaid et al., 2012). However, Rouha et al. (2010) had identified viral small RNAs from six RNA genome viruses. And it had been confirmed that a functional miRNA can indeed be produced during infection without impairing viral RNA replication. The virus could utilize no-canonical DGCR8-independent way to process miRNAs (Shapiro et al., 2010). The basal single-stranded RNA and the terminal loop of miRNA precursors influence the process efficiency (Burke et al., 2014). In fact, the bovine leukemia virus (BLV), a retrovirus with RNA genome, encodes a conserved cluster of miRNAs transcripted by RNA polymerase III (pol III). Thus, the BLV miRNAs avoid the conundrum of genome/mRNA cleavage because only the subgenomic pol III transcripts are efficiently processed into miRNAs (Kincaid et al., 2012). Recently, some new RNA virus original viral miRNAs were found, which played various important roles in virus life cycle and their infectivity (Rouha et al., 2010; Verna and Karyn, 2020) and could also be used as biomarker for early diagnosis (Jayoung et al., 2015; Chen et al., 2016).

*Giardiavirus* (GLV) is a double-stranded RNA virus in the Totiviridae family, which specifically infects trophozoites of the primitive protozoan parasite *G. duodenalis*. It was first identified by Wang and Wang (1986). Previous studies showed that infection of the GLV did not harm the trophozoites (Furfine et al., 1989). The single (+)-strand genome RNA could be used as the GLV template for replication in the trophozoites (Furfine and Wang, 1990). The viral genome length is 6,277 bp. It encodes two proteins, a major 100 kDa capsid protein (Gag) and a minor 190 kDa fusion protein (Gag-Pol) via a-1 ribosomal frameshift. The coding regions of these two proteins share an overlap of 220 nt. The translated region in GLV (+)-strand RNA is flanked by a 367-nucleotide (nt) 5′-UTR and a 301 nt 3′-UTR. The ratio of Gag/Gag-Pol is 120. This proportion is fit to package of virus particles. A virus particle is formed by about 120 Gag proteins and one Gag-Pol coupled with one GLV genome (Wang et al., 1993). Our group has identified a strain of GLV from *G. duodenalis*, which has great genome similarity to the first strain (Cao et al., 2009). Although Wang’s group had used this viral backbone as the vector for transfecting exogenous gene in *G. duodenalis* (Garlapati et al., 2011; Wu et al., 2014), the viral life cycle and self-regulation of host–virus interaction are still unknown.

**FIGURE 1:**
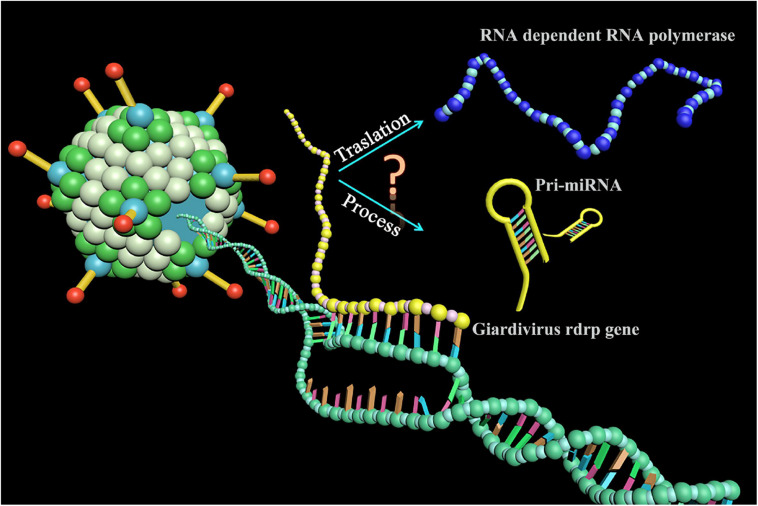
A novel phenomenon for miRNA biogenesis. The transcripts of the *Giardivirus rdrp* gene either translated into RNA-dependent RNA polymerase or processed into miRNA.

In this report, we identified an miRNA-like non-coding RNA (GLV miRNA1) in GLV. Interestingly, the GLV miRNA1 locates in the translated region of viral *rdrp* gene. Knockdown of the GLV miRNA1 could increase the virus copy number in the trophozoites. To our knowledge, this is the first case that an miRNA-like RNA governs the virus copy number and locates in the translated region of a known gene in organism, and this means the transcripts of the (+) strand of the GLV genome either translate into RNA-dependent RNA polymerase (RDRP) or process into miRNA (Figure 1).

## Materials and Methods

### *G. duodenalis* and 293T

The *G. duodenalis* strain (*G. duodenalis* Assemblage A1, GdA1) is preserved in the parasite laboratory of Veterinary Medicine College of Jilin University. And the *Giardiavirus*-free *G. duodenalis* strain was purchased from the ATCC (ATCC^®^ 30957^TM^). The *G. duodenalis* strain maintained in a modified TYI-S-33 medium as previously mentioned (Lu et al., 1990). This *G. duodenalis* is cultured in 10 mL TYI-S-33 culture medium in culture tube at 37°C. The culture tube was tightened to ensure an anaerobic environment, and it was laid at about 15° against the background. The trophozoites were passaged about 2 days for one time. For passaging, the culture tube was incubated at 0°C water for 15 min, and during the incubation, the culture tube was twisted by hands for several times to make sure all the trophozoites cells were dropped from tube wall. The culture medium was centrifuged to collect the trophozoites for future use. If not specially specified, the *G. duodenalis* strain used in this work was virus infected.

The 293T (human embryonic kidney cell) were obtained from the American Type Culture Collection of the Chinese Academy of Sciences (Shanghai, China), which were cultured in Dulbecco minimal essential medium (GIBCO) supplemented with 10% fetal bovine serum (GIBCO) and 100 U/mL penicillin–streptomycin. All cells were cultured in 100 × 20 mm or 60 × 15 mm culture dishes at 37°C in a humidified 5% CO_2_ incubator. All experiments were done using the > 90% confluent cultures. Cells were washed before and after incubation with phosphate-buffered saline (PBS).

### Small RNA Clone and Sequence

The clone of small RNA was based on the previous research (Fu et al., 2005). Briefly, harvesting the exponential phase trophozoites, the small RNAs were enriched with the mirVana kit from Ambion. The enriched small RNA was dissolved in 30 μL RNase-free water. The concentration of RNA was measured by the UV absorbance at 260 nm with Nanodrop (Thermo Fisher Scientific, United States). All small RNAs were polyadenylated at 37°C for 30 min in 50 μL reaction volume with 1.5 μg small RNA and 5 U poly (A) polymerase (Takara, Dalian). The polyadenylated small RNA was purified by phenol/chloroform extraction and ethanol precipitation. A 5′ adapter (5′-CGA CUG GAG CAC GAG GAC ACU GAC AUG GAC UGA AGG AGU AGA AA-3′) was ligated to polyadenylated RNA using T4 RNA ligase (Invitrogen, United States), and the ligation products were recovered by phenol/chloroform extraction followed by ethanol precipitation. Reverse transcription (RT) was performed by SuperScript III kit (Invitrogen, United States). Briefly, the polyadenylated small RNA (10 μL) was incubated with 1 μL (1 μg) of RT primer [5′-ATT CTA GAG GCC GAG GCG GCC GAC ATG-d (T24) M (A,G, or C) N (A,G,C, or T)-3′] and 1 μL dNTP mix (10 mM each) at 65°C for 5 min to remove any RNA secondary structure. The reactions were chilled on ice for at least 1 min. The remaining reagents [5 × buffer, dithiothreitol (DTT), Naseout, SuperScript III] were added as specified in the SuperScript III protocol, and the reaction proceeded for 60 min at 50°C. Finally, the reverse transcriptase was inactivated by 15 min incubation at 70°C. The cDNA amplification was carried out for 25 cycles at a final annealing temperature of 50°C using primers (forward: 5′-ATT CTA GAG GCC GAG GCG GCC GAC ATG T-3′; reverse: 5′-GGA CAC TGA CAT GGA CTG AAG GAG TA-3′). The polymerase chain reaction (PCR) product was separated by 12% polyacrylamide gel with EtBr staining. Then, the gel slices containing DNA with a size about 110–120 bp were excised, and the DNA was eluted into elution buffer [0.5 M NH_4_Ac, 10 mM Mg(Ac)_2_, and 1 mM EDTA] at 37°C and recovered by phenol/chloroform extraction followed by ethanol precipitation. The DNA fragment was directly subcloned into pGEM-T vector (Promega, Japan). Colony PCR was performed using forward primer and reverse primer, and the clones were sequenced with PCR products about 110–120 bp.

### Splint Ligation Northern Blot of GLV miRNA1

Splint ligation was used for the detection of miRNAs according to previous reports (Wu et al., 2014). In short, the trophozoites total RNA (10 μg) was incubated with the GLV miRNA1 bridge oligo (100 pmol) (5′-GAA TGT CAT AAG CGC GGC TGC TGT AAC AGG ACC AGA∼C3 spacer 3′) and the 5′ ^32^P-labled probe (5′-CGC TTA TGA CAT TC-3′) with 1 × Capture buffer (20 mM Tris-HCl, pH 8.0 and 75 mM KCl). The mixture was incubated at 94°C for 1 min, 65°C for 2 min, and 37°C for 15 min. Then, 1 × T4 DNA ligase buffer and 350 U of T4 DNA ligase (Takara, Dalian) were added and incubated at 30°C for 1 h. The final reaction mixture was degenerated at 95°C for at least 5 min before being loaded on to denatured 15% TBE-urea gels, and the membrane was exposed to X-ray films using phosphor imaging screen in intensifier screen at −80°C. Autoradiography was carried out by using a sensitive X-ray film. If not specially specified, the probe was labeled by ^32^P. Typically, each experiment is repeated at least three times.

### Stem–Loop RT–Quantitative PCR Quantity GLV miRNA1 Expression in the Trophozoites

The quantity of GLV miRNA1 expression is based on stem–loop RT–quantitative PCR (qPCR) (Chen et al., 2005). In brief, the trophozoites were counted with a hemocytometer. Approximately 1 × 10^7^ trophozoites suspended cells were pelleted by centrifugation at 1,500 rpm for 5 min, washed with 1 mL Dulbecco PBS without MgCl_2_ and CaCl_2_, and then resuspended in 200 μL of PBS without MgCl_2_ and CaCl_2_. Five-microliter trophozoites solution was heated at 95°C for 5 min and immediately chilled on ice, and then 5 μL RT mixed solution was added (50 nM stem–loop GLV miRNA1 RT primer: 5′-GTC GTA TCC AGT GCA GGG TCC GAG GTA TTC GCA CTG GAT ACG ACC CCG GCT-3′, 1 × RT buffer, 2 mM dNTP, 10 U Rnasein, 100 U M-MLV) (Promega, Japan). The bulk solution was incubated at 42°C for 1 h and 70°C for 5 min and then held at 4°C. The RT reaction products were diluted by 70 times for fluorescent quantitation PCR (Takara, Dalian). The reaction system includes 10 μL Premix Ex Taq (2×) (Probe qPCR), 0.4 μL forward primer [5′-TCT GGT CCT GTT ACA GCA GCC-3′ (10 μM)], 0.4 μL reverse primer [5′-CAG TGC AGG GTC CGA GGT A-3′ (10 μM)], 0.8 μL fluorescent probe [5′-FAM-CAG CCG GGG TCG TAT CCA GTG CGA A-3′ BHQ (10 μM)], and 8.4 μL RT product. The reactions were incubated at 95°C for 10 min, followed by 40 cycles at 95°C for 15 s and 60°C for 1 min. The threshold cycle (CT) was defined as the fractional cycle number at which the fluorescence passed the fixed threshold. TaqMan CT values were converted into absolute copy numbers using a standard curve from synthetic GLV miRNA1. All reactions were run in triplicate independently.

### Virus Copy Number Determination

The transcript amounts of capsid mRNA were used to reflect the genomic RNA levels of GLV (forward: 5′-ACG TAC ACG TCC AAC TCT CCA TTA-3′; reverse: 5′- CTG CGT GAA GAC TTT CGA TGAT-3′; probe in the region: 5′-FAM-CAC ACG TGG CGG AGC CTC ACAT-3′ BHQ). And the glyceraldehyde-3-phosphate dehydrogenase (GAPDH) transcript amounts were used to represent *G. duodenalis* amounts (forward: 5′-CGA CCC CTT CAC GGA CTGT-3′; reverse: 5′-TCC TCG GGC TTC ATA GAC TTG-3′; probe in the region: 5′-FAM-CGG CAC CAT CGC CCA CTC TGA-3′ BHQ). The ratio of transcript amounts between capsid and GAPDH can be used as the virus copy number in each trophozoite. Briefly, approximate 1 × 10^7^ trophozoites were suspended in 200 μL PBS without MgCl_2_ and CaCl_2_. 5 μL of the mixture was heated at 95°C for 5 min, and immediately chilled on ice and was subjected to RT after adding to the RT reaction mixture (0.1 μM oligo dT18 primer, 1 × RT buffer, 1 mM dNTP, 10 U Rnasein, 100 U M-MLV). The 20 μL reactions were incubated at 42°C for 1 h and 70°C for 5 min. Real-time PCR was performed using a standard TaqMan PCR kit protocol and analyzed by CFX_Manager. Typically, each experiment is repeated at least three times separately in succession.

### Primer Extension for Pre-GLV miRNA1

Primer extension was performed to verify the existence and the length of pre-GLV miRNA-1 according to previous description (Rice et al., 2014). Briefly, 24 nt 5′ ^32^P-labled DNA primer (5′-CCT GGT TGG CTT GTT AAG GAC CAC-3′) was used for RT reactions. Trophozoites RNAs (10 μg) and 5′ ^32^P-labeled primer (1 pmol) were denatured for 3 min at 95°C and then cooled down on ice for 3 min, annealed at 43°C for 10 min in 50 mM Tris-HCl (pH 8.0), 50 mM KCl, 5 mM MgCl_2_, 5 mM DTT, and 50 ng/mL gelatin. The primer extension reaction was carried out in the presence of 10 U of AMV reverse transcriptase (Promega, Japan), 40 U of RNasin (Promega, Japan), and 1 mM dNTP at 43°C for 30 min. Template RNA was digested in 250 ng/mL RNase A at 43°C for 10 min. The extension products were resolved by electrophoresis on denatured 15% TBE-urea gels, and the membrane was exposed to X-ray films using phosphor imaging screen in intensifier screen at −80°C. Autoradiography was carried out using a sensitive X-ray film. All reactions were run in triplicate independently.

### *In vitro* Assay of GlDcr Digestion of Pre-GLV miRNA1

The pre-GLV miRNA1 DNA was RT-PCR amplified from virus genome with primers (T7 forward: 5′-TAA TAC GAC TCA CTA TAG GAT GGG ATC CTA CGC AGG AAA G-3′, T7 reverse: 5′-CCT CCT AAG CCG GTA CAG TTA AC-3′), and subcloned into pMD-18 T vector (Promega, Japan) and sequenced. The linearized correct vector was used as template for *in vitro* transcription of pre-GLV miRNA1. *In vitro* transcription was carried according to manufacturer protocol (Promega, Japan): template DNA (1 μg total), RiboMAX^TM^ Express T7 2X buffer (10 μL), T7 Express (2 μL), and nuclease-free water were added to 20 μL volume and then mixed gently and incubated at 37°C for 30 min. For the GlDcr digestion assay, approximately 20 μg pre-GLV miRNA1 RNA was incubated with 8 U of the commercial GlDcr (Powercut Dicer, Finnzyme) for 16 h at 37°C. The reaction solution was purified by phenol–chloroform extraction. The purified product was examined by Splinted ligation as previously reported (Wu et al., 2014). Typically, this experiment is repeated at least three times in succession.

### Antisense Inhibitor for GLV miRNA1

Antisense-GLV miRNA1 (5′-2′-O-methyl CGG CUG CUG UAA CAG GAC CAG A-3′) and a scrambled control sequence (control RNA: 5′-UAC UCU UUC UAG GAG GUU GUG A-3′) were synthesized by TaKaRa. Antisense-GLV miRNA1 (1 μg) or the control sequence (1 μg) was transfected into trophozoites by electroporation. Briefly, trophozoites were harvested at 1,000 rpm in 5 min at 4°C and resuspended in a density of 1 × 10^7^/mL. After being washed in cooled PBS twice, then the trophozoites were resuspended in 300 μL electroporation buffer [10 mM K_2_ HPO_4_-KH_2_PO_4_ (pH7.6), 25 mM HEPES (free acid), 120 mM KCl, 0.15 mM CaCl_2_, 2 mM EGTA, 5 mM MgCl_2_, 2 mM ATP, 4 mM glutathione]. The synthetic antisense-GLV miRNA1 or control inhibitor was added into cell suspension, and then the reactions were incubated on ice for 10 min and performed electroporation using a Bio-Rad Gene Pulser X cell (voltage: 450 V, capacitance: 500 μF, resistance: ∞). Cells were incubated on ice for 15 min, added to prewarmed culture medium, and then were collected 6 h after transfection and subjected to RT-qPCR assay using the above specific primers to quantify *Giardivirus* capsid transcript as the copy number of virus. This experiment is repeated at least three times in succession.

### Virus Mimic Construction

*Giardivirus* vector pac631 was a gift from Wang CC. The transcript of this vector can be duplicated when it was electroporated into the virus containing *G. duodenalis* trophozoites. The pac631 was digested by *Hin*dIII/*Xho*I, and the backbone was recovered. Then the SNAP gene was constructed into the pac631 backbone. The recombined plasmid was termed as pac631-SNAP. The linearized pac631-SNAP vector was used as template for *in vitro* transcription of virus mimic. These virus mimics were transfected into trophozoites by electroporation. The electroporation parameter sets were same as previously described. After 72 h, the trophozoites were harvested, and the SNAP substrate was added into trophozoites suspension at 37°C for 30 min. And then the trophozoites were analyzed by laser confocal microscope and flow cytometry. The flow cytometry is repeated at least three times in succession.

### Scanning Electron Microscope of the *G. duodenalis* Trophozoites

Trophozoites were pelleted and postfixed in 1% osmic acid in 0.1 M sodium cacodylate for 1 h. Ultrathin sections were then stained with lead citrate and uranyl acetate, dehydrated in ethanol, critical point dried with CO_2_, and sputter-coated with gold. Specimens were examined using a JEOL JSM-5500LV scanning electron microscope (SEM).

### Target Prediction

Targets of GLV miRNA1 were predicted with miRanda (John et al., 2004) on the entire genome of GLV. Because the function of the GLV miRNA1 was related to the virus copy number, we focused our target’s identification on GLV genome strand (+), which controlled the replication process of virus (score threshold = 120, energy threshold = 20 kcal/mol, and scaling factor = 4).

### Luciferase Assays

Luciferase assays were performed as recommended by the manufacturer (Promega, Japan). In the target confirming experiment, the positive control vector pmirGLO-2x-RE-GLV miRNA1 was constructed by incorporating two copies of perfectly artificial targets complementary to GLV miRNA1 into the 3′-UTR of luc2 in pmirGLO. And the vectors (pmirGLO-2x-5′-UTR, pmirGLO-2x-5′-gag and pmirGLO-2x-3′-UTR) containing the putative GLV miRNA1 targets were constructed by incorporating two copies of 5′-UTR, 5′-gag, and 3′-UTR target sequence, respectively, into the 3′-UTR of luc2 in pmirGLO. PmirGLO-2x-RE-GLV miRNA1, pmirGLO-2x-5′-UTR, pmirGLO-2x-5′-gag, pmirGLO-2x-3′-UTR, or pmirGLO vector were, respectively, cotransfected into 293T cell with synthetic GLV miRNA1 (5′-phos-UCU GGU CCU GUU ACA GCA GCC G-3′) by Lipofectamine 2000 reagent (Invitrogen, United States) according to the manufacturer protocol. Luciferase assays were performed after 32 h of transfection using Dual-Luciferase Reporter Assay System (Promega, Japan) and Pharmingen Monolight 3020 luminometer. Each value of firefly luciferase (Rr) was normalized to the Renilla luciferase value (Pp). Each value was an average of three independently transfections with the standard indicated deviation.

### Analysis of the GLV miRNA1 Cleavage Efficiency on the Putative Targets

Real-time PCR based on SYBR–green I fluorescence (Roche, Switzerland) was employed to analyze the miRNA cleavage efficiency on the potential targets. Primers were designed as follows: 5′-UTR target (forward: 5′-AGT GCC AGG CCA TTA CCT TCC; reverse: AAG CGT GGC GAA GTC TAG CAG C-3′), 5′-gag target (forward: 5′-CAG GAG TCT CAT TTC CCC TCC-3′; reverse: 5′-GAA GCC GTA CGG GTT TAA CGG-3′), 3′-UTR target (forward: 5′-ATT ACC GCA CAC GAT CGG TGG; reverse: 5′-TAC GCT GCC TCC TAC AGG CAA C-3′), red fluorescence protein (RFP, reference) (forward: 5′-GTG CAG CAG GAC TCC TCC CT-3′; reverse: 5′-CGG GCT TCT TGG CCT TGT AC-3′). In short, four RNA strands (pc631-RFP-5′-UTR, pc631-RFP-5′-Gag, pc631-RFP-3′-UTR, pc631-RFP) were achieved by *in vitro* transcription. And each RNA (2 μg) was cotransfected with 25 pmol synthetic GLV miRNA1 into 293T cell. Real-time RT-PCR assay was performed after 6 and 12 h, respectively, to evaluate the cleavage efficiency of GLV miRNA1 on the 5′-UTR, 5′-Gag, and 3′-UTR targets real time, and the pc631-RFP was used as the reference gene by the 2−ΔΔCT method. Typically, each experiment is repeated at least three times in succession.

## Results

### Clone of Potential GLV Small RNA

**FIGURE 2:**
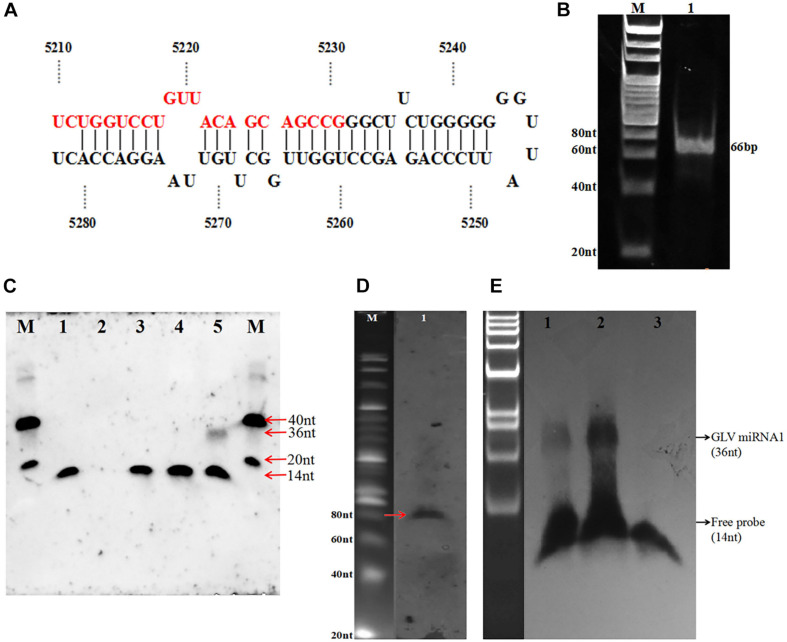
Identification of GLV miRNA1. **(A)** The hairpin structure sequence containing GLV miRNA1. **(B)** The polymerase A–based RT-PCR of GLV miRNA1, 1: DNA ladder, 2: amplicons of GLV miRNA1. **(C)** Splinted ligation Northern blot of GLV miRNA1, M: oligo = 40 nt (5′-CGT ACG TAT ATA TTT ATG GCG GCG CGA TGC TAC TCT CTC G-3′ DIG) + 20 nt (5′-CGT ACG TAT ATA TTT ATG GC-3′ DIG), 1: probe (5′-phosCGC TTA TGA CAT TC-3′ DIG 14 nt), 2: bridge (5′-GAA TGT CAT AAG CGC GGC TGC TGT AAC AGG ACC AGA-3′ 36 nt), 3: probe and bridge, 4: probe + bridge + *G. duodenalis* RNA (virus-free), 5: probe + bridge + *G. duodenalis* RNA(with virus). **(D)** Existence of pre-GLV miRNA1 by primer extension M: 20 bp marker (TaKaRa), 1: products of primer extension. **(E)** GlDcr is required for processing the mature GLV miRNA1, 1: *G. duodenalis* trophozoites total RNA, 2: pre-GLV miRNA1 RNA digested with GlDcr, 3: pre-GLV miRNA1 RNA digested without GlDcr.

In order to explore the new potential miRNA of the GLV infectious *G. duodenalis*, we constructed a cDNA library of size-fractionated small RNA from the GLV infectious *G. duodenalis* trophozoites. The library was cloned into a pGEM-T vector and sequenced. Fifty-one eligible sequences were obtained (Table 1). Most of those sequences were the half tRNA of the *G. duodenalis*, and some are from the unannotated of *G. duodenalis* genome. Surprisingly, one of those small RNAs is from GLV genome. The sequence is 5′-UCU GGU CCU GUU ACA GCA GCC G-3′ (22 nt) (Figure 2A). The small RNA codes in the (+) strand of GLV from nucleotides 5,210 to 5,231, which is a translated region of virus RNA-dependent RNA polymerase (*rdrp*) gene. Interestingly, the result from the viral miRNA prediction software (VMir)(3) shows that the virus genome has several potential miRNA hairpins. And the rank 1 score (173.2) hairpin structure is from nucleotides 5,212 to 5,280 (Supplementary Figure 1), ΔG = −47.31 kcal ⋅ mol^–1^, and the 22 nt small RNA is exactly in the 5′ arm of this hairpin structure.

**TABLE 1:** A serial small RNA sequence of GLV.

Number	Sequence (5′–3′)	Size (nt)	No. of clones	Location
1	GCGTTCCCATGACGGTCGCAGAC	23	4	*Giardia* genome
2	TGGGGTGTGGTGCAGCGGGAGCACAG	26	5	Gln-tRNA
3	TCTGGTCCTGTTACAGCAGCCG	22	10	*Giardiavirus* genome
4	AGGAAGAGTCAGTTTGTAGT	20	3	*Giardiavirus* genome
5	TGACGAAGTTTGTCGTATTTTGAAT	25	12	GlsR1 noRNA
6	CTAAAGTAAATGCCTCCC	18	1	*Giardia* genome (unannotation)
7	GTCTTAGTAGAGTGAATGGTCCCGTTC	27	5	*Giardia* genome
8	TTGATCCAGAAGTAAACATGCAGG	24	6	*Giardia* genome
9	CTGAAGTCCGAGAAGTCCTG	20	4	*Giardia* genome
10	CCTGTAGGAGGCAGCGTACGAAGGGGTC	28	1	*Giardiavirus* genomic

### Identification of GLV miRNA

To verify this putative GLV, miRNA is a specific product in GLV or a random digest produced sequence, we used the complementary sequence of this potential miRNA to probe its expression in GLV infectious trophozoites by DNA ligase–based splitting Northern blot with size-fractionated small RNA (<200 nt) (Wu et al., 2014). The result showed an unambiguous ∼36 nt band in the GLV infectious trophozoites sample. However, no any specific bands were detected in other samples, including GLV-free trophozoites sample (Figure 2C). It suggests this sequence is a processed product of GLV genome, but not a random degraded sequence. This sequence is termed as GLV miRNA1.

### Precursor of GLV miRNA1 Probe by Primer Extension

Although we have predicted a hairpin structure containing the GLV miRNA1 in the 5′ arm, the existence of the GLV miRNA1 precursor is uncertain. We designed a DNA oligo (5′-CCT GGT TGG CTT GTT AAG GAC CAC-3′ (24 nt) as the primer to explore the GLV miRNA1 precursor by primer extension. As anticipated, an approximate 80 nt extension product was recovered in the GLV infectious trophozoites sample (Figure 2D). It showed that the precursor of GLV miRNA1 is also a processed product of the GLV genome.

### *In vitro* Assay of GlDcr Digestion of Pre-GLV miRNA1

Generally, the miRNA process needs the Drosha to produce the pre-miRNA and then processed to mature miRNA by Dicer. However, there is no any homolog of Drosha in the *Giardia* genome. The GIDcr is the first crystallized Dicer from the *G. duodenalis*. It could directly splice the long up to 155 bp duplex RNA into 25–27 bp small RNA *in vitro* (MacRae et al., 2006). But there is no any information about the GIDcr to process the longer RNA with stem loop structure. To explore how the excess GIDcr deals with the GLV RNA, the RNA of the GLV infectious trophozoites was incubated with GIDcr *in vitro*. However, the result did not show any increase of GLV miRNA1 quantity, but when the *in vitro* transcripted (+) strand GLV genome was used as the substrate of GIDcr, the amount of GLV miRNA1 was extensively increased (Figure 2E). Unequivocally, all the results confirmed that GLV miRNA1 was a specific product of GLV genome. And the GIDcr can process the GLV RNA (+) strand into GLV miRNA1 by itself.

### Ascertainment of GLV miRNA1 Copy Number

After confirming the GLV miRNA1 was a specific product, the expression of GLV miRNA1 was detected by polymerase A based RT-PCR (Fu et al., 2006). The result showed that a special band about 66 bp was obtained (Figure 2B). Then the copy number of GLV miRNA1 in each GLV-infected trophozoite was quantified by stem–loop RT–qPCR (Chen et al., 2005). We used a synthesized GLV miRNA1 as control to construct the standard curve with *R* = 0.974 (Supplementary Figure 2), which could be used to confirm copy number of GLV miRNA1. Based on the standard curve, we confirmed that there were about 500 copies of GLV miRNA1 in each GLV-infected trophozoite.

### Decreased GLV miRNA1 Will Increase Virus Copy Number in Trophozoites

**FIGURE 3:**
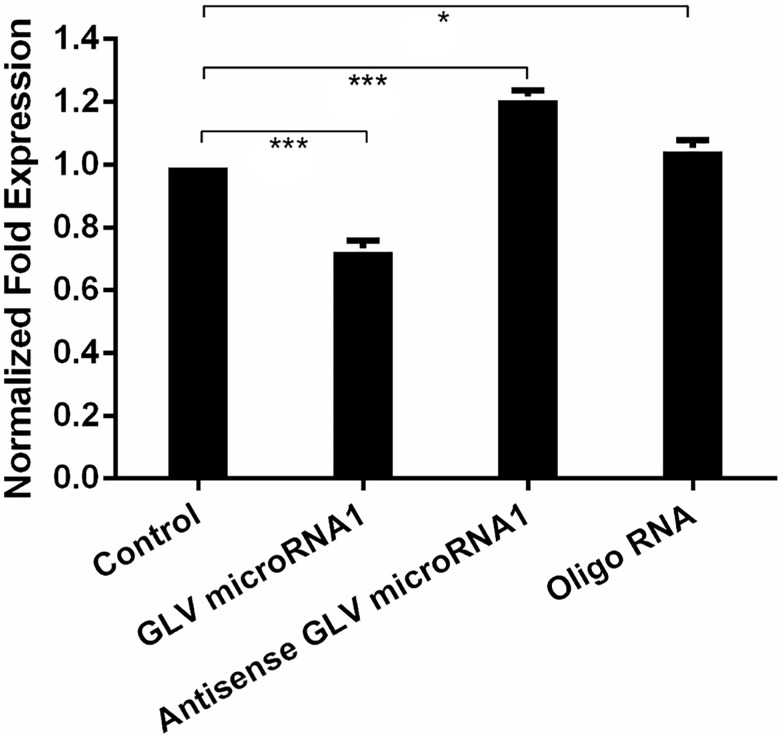
Decreased GLV miRNA1 will increase virus copy number in trophozoites. Control: no RNA was transfected into trophozoites; GLV miRNA1: GLV miRNA1 was transfected into trophozoites; Antisense GLV miRNA1: antisense GLV miRNA1 was transfected into trophozoites. Oligo RNA: a scrambled control was transfected into trophozoites. Error bars represent 99% confidence intervals. The difference was significant [unpaired *t*-test: *p* < 0.001 (GLV miRNA1), *p* < 0.001 (antisense GLV miRNA1), *p* = 0.0214 (oligo RNA); *N* = 12]. *p < 0.05, ***p < 0.001.

In order to explore the role of GLV miRNA1, we knock down the GLV miRNA1 by transfecting completely synthesized antisense RNA of GLV miRNA1 into trophozoites. However, there was no obvious change in *G. duodenalis* life cycle, motility, proliferation, and pathogenesis. Interestingly, we found the antisense RNA could increase virus copy number about 30% in each trophozoite; meanwhile, the GAPDH expression level was used as inner reference to ensure the reliability of the result. And transfecting the synthesized GLV miRNA could decrease the copy number less than 30% with a scrambled RNA as the control (Figure 3).

### The Construction of the Visual Virus Mimics

**FIGURE 4:**
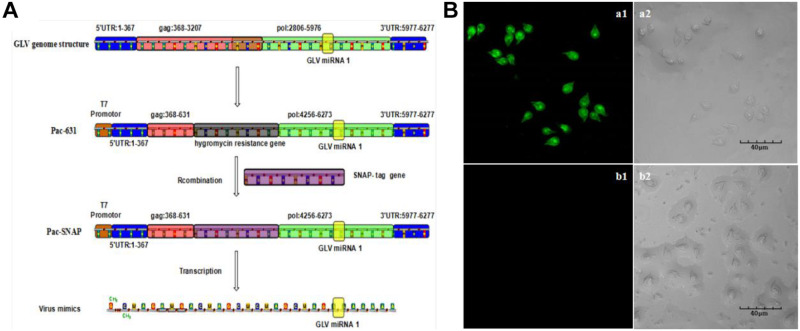
The construction of the visual virus mimics. **(A)** The scheme of the virus mimics construction. **(B)** The virus mimics were transfected into the *G. duodenalis* trophozoites. (a1) The laser confocal imaging of *Giardivirus* infectious *G. duodenalis.* (a2) The bright-field imaging of *Giardivirus* infectious *G. duodenalis.* (b1) The laser confocal imaging of *Giardivirus*-free *G. duodenalis.* (b2) The bright-field imaging of *Giardivirus*-free *G. duodenalis*.

Although our group had developed a green fluorescence protein (GFP)–based visual virus (Liu et al., 2005), the GFP fluorescence intensity is dependent on the oxygen, and the culture environment of *G. duodenalis* is anaerobic. Therefore, the imagination process is very tedious. SNAP-tag is a 182-residue (19.4 kDa) self-labeling protein tag that can be fused to any protein of interest. The complexes of the SNAP tag and the substrate could be excited to emit green fluorescence in complicated condition, including anaerobic environment. The most part of the *Giardiavirus* genome was deleted by C. C. Wang group to construct the pac631 vector, which retained only the regulated elements for duplication and exogenous expression (Yu et al., 1996). The transcripts of the pac631 can subsist in the *Giardiavirus* infectious trophozoites for several decades’ generation with the help of wild-type *Giardiavirus* RDRP. To confirm the previous results that knockdown of GLV miRNA1 will increase the copy number of virus, we just substituted the resistance gene with SNAP gene to express the SNAP protein (pac631-SNAP). The transcripts of pac631-SNAP will be replicated in the *Giardiavirus* infectious trophozoites accompanied by the *Giardiavirus* reproduction. And it could be translated into SNAP-tagged protein, which could be visible by interaction with the SNAP substrate along with the emission of green fluorescence. Thus, the fluorescence intensity could be used to represent the amount of the virus (mimics virus). The results showed that, after 20 times passage, the mimic virus–contained *Giardiavirus* infectious trophozoites still present high green fluorescence (Figure 4A). However, in the *Giardiavirus*-free trophozoites, without the help of wild-type *Giardiavirus* RDRP, the transcripts of pac631-SNAP cannot be replicated; therefore, there is no any green fluorescence (Figure 4B).

### Virus Amount Assess With Mimics Virus

**FIGURE 5:**
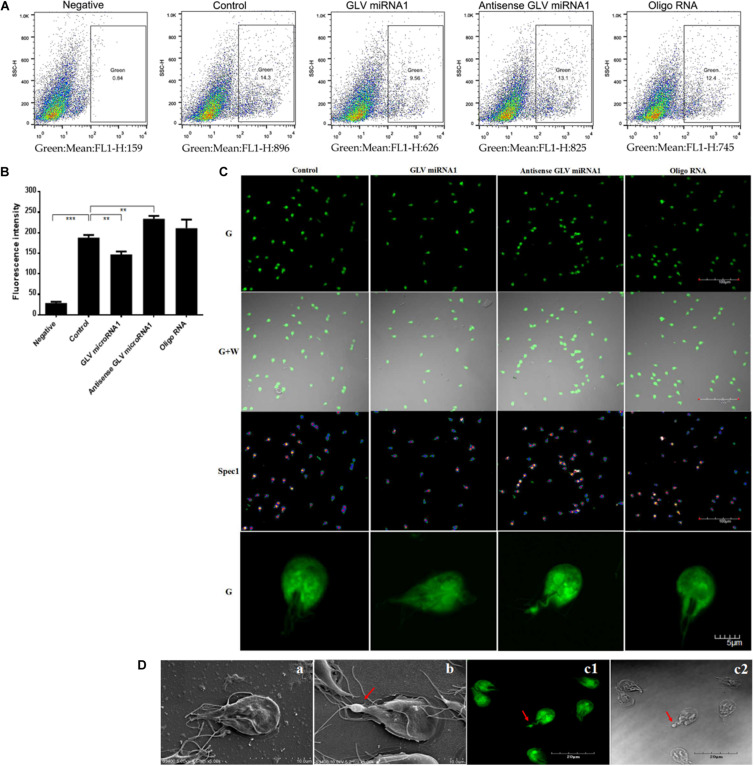
The functions of GLV miRNA1. **(A)** The flow cytometry results of trophozoites transfected by mimic virus, Negative: trophozoites transfected by mimic virus, Control: trophozoites transfected by mimic virus plus SNAP substrate, GLV miRNA1: trophozoites transfected by mimic virus plus SNAP substrate and GLV miRNA (5μM), Antisense GLV miRNA1: trophozoites transfected by mimic virus plus SNAP substrate and antisense GLV miRNA (5 μM), Oligo RNA: trophozoites transfected by mimic virus plus SNAP substrate and scramble RNA (5 μM). **(B)** flow cytometry results. Error bars represent 99% confidence intervals. The difference was significant [unpaired *t-*test: *p* < 0.001 (negative), *p* = 0.0019 (GLV miRNA1), *p* = 0.0011 (antisense GLV miRNA1), *p* = 0.1466 (oligo RNA); *N* = 3]. **p < 0.01, ***p < 0.001. **(C)** Laser confocal microscope of trophozoites transfected by mimic virus, G: green; G + W: merge of green and white; Spec1: spectrum. **(D)** The bulge in the tail part of *G. duodenalis*; (a) the typical *Giardivirus*-free *G. duodenalis*; (b) the typical *Giardivirus* infectious *G. duodenalis*, the bulge location was consistent with the laser confocal microscope result, and the arrow pointed to the bulge of tail; (c1) the typical laser confocal imaging of *Giardivirus* infectious *G. duodenalis*, the arrow pointed to the bulge of tail; (c2) the bright-field imaging of *Giardivirus* infectious *G. duodenalis*, the arrow pointed to the bulge of tail.

Because the mimics could reflect the amount of the wild-type *Giardiavirus*, the mimic virus transfected *Giardiavirus* infectious trophozoites could be used as the fluorescence reporter to assess the *Giardiavirus* amount. By this reporter, we can reconfirm the function of the GLV miRNA1 with flow cytometry and laser scanning microscope. The results showed that the transfection by GLV miRNA1 could decrease the proportion of green fluorescence–positive trophozoites, and the antisense GLV miRNA1 could increase the positive compartment (Figure 5A). And the visual observation under the laser scanning confocal microscope showed that, in contrast to the control group, the number of trophozoites with high fluorescence intensity rose in the antisense GLV miRNA1 group, and the GLV miRNA1 would reduce the proportion of trophozoites with high fluorescence intensity (Figure 5B). Careful observation results showed that some trophozoites have a green light spot in the tail part. The amount of green light spots–contained trophozoites increased in the antisense GLV miRNA1 group. The SEM results showed that most *Giardiavirus* infectious trophozoites actually have a bulge in the tail part (Figure 5C). This implied that both of the green light spot from the laser scanning confocal microscope and the tail bulge from the SEM would be the collection region of *Giardiavirus* (Figure 5D). And in summary, all the above results suggest that the GLV miRNA1 directly governs the virus copy number.

### Potential Target of GLV miRNA1

**FIGURE 6:**
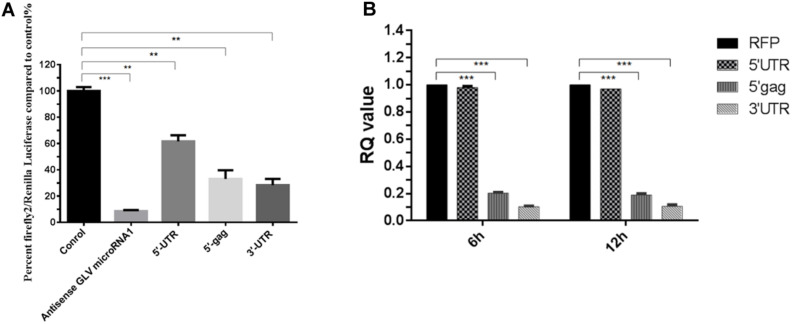
The putative targets of GLV miRNA1. **(A)** Luciferase assays of the predicted GLV miRNA1 targets; Error bars represent 99% confidence intervals. The difference was significant [paired-samples *t-*test: *p* = 0.0002 (antisense GLV miRNA1), *p* = 0.0011 (5′-UTR), *p* = 0.010 (5′-gag), *p* = 0.002 (5′-UTR); *N* = 12]. **(B)** Real-time PCR analysis of the GLV miRNA1 cleavage efficiency on the putative targets, real-time PCR results at 6 and 12 h posttransfection. RFP is the reference gene, and 5′-UTR, 5′-gag, and 3′-UTR represent the putative GLV miRNA1 targets. Error bars represent 99% confidence intervals. The difference was significant [unpaired-samples *t-*test: *p* = 0.377 (5′-UTR); *p* < 0.001 (5′-gag); *p* < 0.001 (5′-UTR); *N* = 12]. **p < 0.01, ***p < 0.001.

Although GLV miRNA1 could govern the copy number of virus in host cell, the target of GLV miRNA1 is still unknown. We used the Miranda to predict the target of GLV miRNA1 (John et al., 2004). The result showed that there were three potential targets, 5′ Gag, 5′-UTR, and 3′-UTR of *Giardiavirus*. And then the luciferesence assay was used to verify the real target of GLV miRNA1. The results showed that 5′ Gag and 3′-UTR could dramatically decrease (about 70%) the expression of reporter gene. And the 5′-UTR could also decrease the expression of reporter gene about 20% (Figure 6A). In order to verify the true targets that could be cleaved by GLV miRNA1, we performed the analysis of the GLV miRNA1 cleavage efficiency on the three potential targets by real-time RT-PCR. The results showed that the target (5′ Gag, and 3′-UTR) sequences were degraded by GLV miRNA1, whereas the 5′-UTR sequence could not be cleaved by GLV miRNA1 (Figure 6B). Thus, we presumed that the 5′ Gag and 3′-UTR sequences had more contributions to the function of GLV miRNA1.

## Discussion

With mounting evidences, the RNA viruses can encode miRNAs (Chirayil et al., 2018; Duy et al., 2018). West Nile virus, single-stranded RNA genome virus, encodes an miRNA in its 3′-UTR, which has a number of stem loops (Hussain et al., 2012). The deep sequence strategy was also utilized to characterize the dengue virus–coding miRNAs, which could facilitate the replication of virus (Hussain and Asgari, 2014). *Giardiavirus* is a double-stranded RNA virus of Totiviridae family. The viral genome length of *Giardiavirus* is 6,277 bp. *Giardiavirus* can specifically infect *G. duodenalis*, but it is not lethal to its host. It was previously reported that there is no difference between the virus-infected *G. duodenalis* and virus-free *G. duodenalis* in life cycle and pathogenicity (White and Wang, 1990). Recently, our group successfully constructed a *Giardiavirus*-infected *G. duodenalis* (named VIG strain) based on *Giardia lamblia* WB strain (ATCC30957) and found that VIG strain trophozoites proliferated significantly faster than virus-free *G. lamblia* WB strain. Moreover, the cyst-forming rate of VIG strain was significantly lower than that of *G. lamblia* WB strain (data unpublished). This indicated that deep research on *Giardiavirus* would contribute to explore the pathogenicity of *G. duodenalis*. The genome of *Giardiavirus* has several unique characters including an internal ribosome entry site (IRES) in 5′-UTR. Most interestingly, the IRES also occupied the first part of the capsid gene translated region (264 nt). This is the first identified IRES located in the translated region. So this cistron has two functions: coding capsid protein and enhancing translation efficiency. The replication and transcription of this virus are controlled by RDRP, so it has a similar architecture in its 5′ end and 3′ end, respectively (Supplementary Figure 3). We have the point that it could be recognized by RDRP, and the difference between replication and transcription mechanism should be achieved by a region beside this structure. Wang’s group has developed a *Giardiavirus*-based exogenous gene expression vector, which contained not only the part of 5′ translated region of *gag*, but also some 3′ translated region of *rdrp* to elevate the property of vector (Yu et al., 1996). The GLV miRNA1 is also in the 3′ translated region of *rdrp*. Although several miRNAs have been identified in the *G. duodenalis*, there are still some questions about the process of miRNA maturation. No homolog of Drosha was found in the *Giardia* genome. The *in vitro* GlDcr digestion assays implied that Dicer itself could process the RNA of *in vitro–*transcripted (+) strand *Giardiavirus* genome into mature miRNA. And the *in vivo* pathway still needs more elaborate explorations. What is surprising is that the miRNA locates in the 3′ region of *rdrp* translated region, which means this region has at least two functions for this virus: coding RDRP and processing to miRNA. This is a novel phenomenon for miRNA biogenesis. There must be some balance between the translation into RDRP and the process into GLV miRNA1-like Kaposi sarcoma–associated herpesvirus (Lin and Sullivan, 2011). The detailed mechanism needs more ingenious investigations. First, we used complementary sequence to inhibit the functions of GLV miRNA1; the results showed that inhibiting the role of GLV miRNA1 would increase the *Giardiavirus* copy number. To reconfirm the result, we constructed a mimic virus based on the pac631 vector to visualize the virus. And the flow cytometry and laser scanning confocal microscope results showed that GLV miRNA1 is involved in the government of virus copy number by participating in the replication of virus.

When predicting GLV miRNA1 target sequence, we found it had three potential targeting regions: 5′-UTR, 5′-Gag, and 3′-UTR. The luciferase assays results showed that the 5′-Gag and 3′-UTR contained reporter decrease of about 70% and 5′-UTR only about 20%. GLV miRNA1 cleavage efficiency assay indicated that the GLV miRNA1 could not cleave the 5′-UTR sequence. So the target of GLV miRNA1 should be the 5′-Gag and 3′-UTR. What was more interesting was the fact that the 3′-UTR target sequence was adjacent to the RDRP-recognized structure in its 3′ end. Thus, the GLV miRNA1 should play a function in the 3′-UTR to control the replication of virus, although the direct evidence remains for further investigation.

In summary, we have identified a new miRNA from *Giardiavirus*, which is coded in the translated region of a known gene. And the GLV miRNA1 could govern the virus copy number in its host by targeting RDRP-recognized sequence. Overall, GLV miRNA1 is the first miRNA found to be located in the coding region of a gene, and this novel phenomenon for miRNA biogenesis may further broaden researchers’ understanding of the way of generating non-coding RNA. The balance between coding miRNA and translated proteins may be a new way for regulating gene expression.

## Data Availability Statement

The original contributions presented in the study are included in the article/Supplementary Material, further inquiries can be directed to the corresponding authors.

## Author Contributions

PG and XiaL designed the research. PG, XiaL, WW, and CL conducted the research. PG, LC, and PZ wrote the manuscript. XinL, PG, CL, and BR analyzed the data. XZ and JL directed the project. All authors contributed to the article and approved the submitted version.

## Conflict of Interest

The authors declare that the research was conducted in the absence of any commercial or financial relationships that could be construed as a potential conflict of interest.
