# Supplementary material for: A Novel MicroRNA From the Translated Region of the Giardiavirus rdrp Gene Governs Virus Copy Number in Giardia duodenalis
Source: Front Microbiol. 2020 Nov 23;11:569412. doi: 10.3389/fmicb.2020.569412 (PMC7719678; doi:10.3389/fmicb.2020.569412)

**Figure supplement 1.**

VMir analysis of the Giardiavirus genome; shown are all hairpins that achieved a VMir score of 50 or above. Hairpins are plotted according to genomic location and VMir score. The highest score hairpin is marked (score of 173.2), which contain we identified GLV mRNA1 sequence.
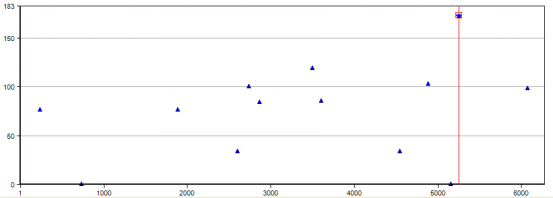


**Figure supplement 2 .**

Standard curve of GLV microRNA1 quantity.○: standard; ×: GLV miRNA1 amount; the *R=*0.974. The data represent mean ± SEM of one of 3 independent experiments, n = 3


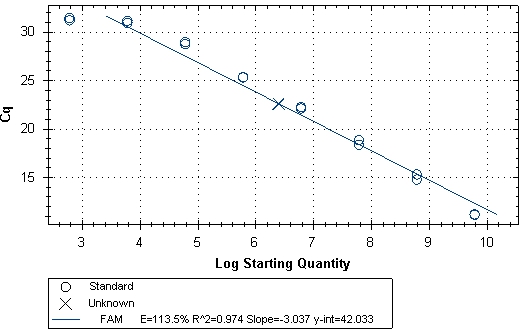


**Figure supplement 3**

The secondary structure architecture. The 5' architecture of giardivirus(A) is similar to the 3 ' architecture (B), which imply they all could interact with RDRP.


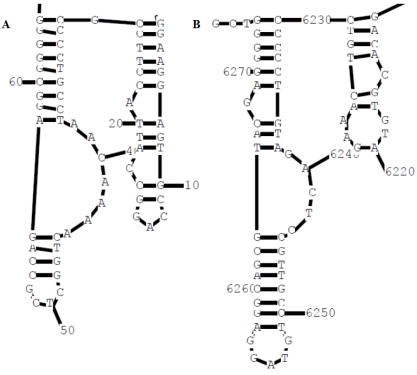

Supplement: Supplementary file 1 [file Data_Sheet_1.doc]
